# Supplementary material for: Cell cycle-dependent phosphorylation regulates RECQL4 pathway choice and ubiquitination in DNA double-strand break repair
Source: Nat Commun. 2017 Dec 11;8:2039. doi: 10.1038/s41467-017-02146-3 (PMC5725494; doi:10.1038/s41467-017-02146-3)

a

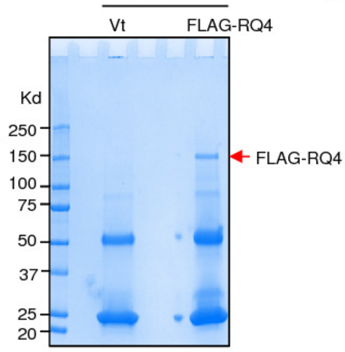

b

Ser89: AATK*SPQPTPGR

Seq	#	b	y	+1
A	1	72.0	-	12
A	2	143.1	1219.6	11
T	3	244.1	1148.5	10
K	4	372.2	1047.5	9
*S	5	539.2	919.4	8
P	6	636.3	752.4	7
Q	7	764.3	655.3	6
P	8	861.4	527.3	5
T	9	962.4	430.2	4
P	10	1059.5	329.2	3
G	11	1116.5	232.1	2
R	12	-	175.1	1

Ser251: VG*SPQPSSSGGEK

Seq	#	b	y	+1
V	1	100.1	-	13
G	2	157.1	1197.5	12
*S	3	324.1	1140.4	11
P	4	421.1	973.4	10
Q	5	549.2	876.4	9
P	6	646.3	748.3	8
S	7	733.3	651.3	7
S	8	820.3	564.2	6
S	9	907.4	477.2	5
G	10	964.4	390.2	4
G	11	1021.4	333.2	3
E	12	1150.4	276.1	2
K	13	-	147.1	1

c

Ser27: RP*SQDDVEAAPEETR

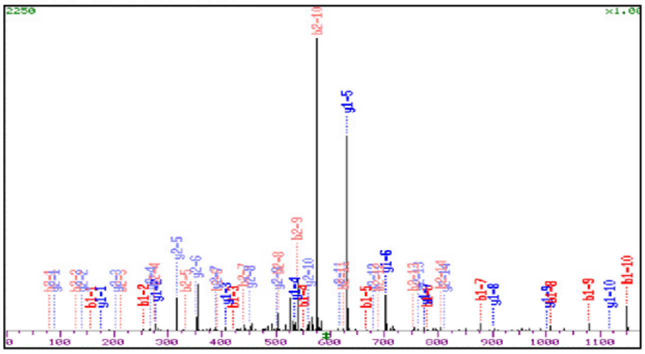

Seq	#	b	y	+1
R	1	157.1	-	15
P	2	254.2	1623.6	14
*S	3	421.2	1526.6	13
Q	4	549.2	1359.6	12
D	5	664.2	1231.5	11
D	6	779.3	1116.5	10
V	7	878.3	1001.5	9
E	8	1007.4	902.4	8
A	9	1078.4	773.4	7
A	10	1149.5	702.3	6
P	11	1246.5	631.3	5
E	12	1375.5	534.2	4
E	13	1504.6	405.2	3
T	14	1605.6	276.1	2
R	15	-	175.1	1

Seq	#	b	y	+2
R	1	79.1	-	15
P	2	127.6	812.3	14
*S	3	211.1	763.8	13
Q	4	275.1	680.3	12
D	5	332.6	616.3	11
D	6	390.1	558.8	10
V	7	439.7	501.2	9
E	8	504.2	451.7	8
A	9	539.7	387.2	7
A	10	575.2	351.7	6
P	11	623.8	316.1	5
E	12	688.3	267.6	4
E	13	752.8	203.1	3
T	14	803.3	138.6	2
R	15	-	88.1	1

d

	Ser89	Ser251
H. sapiens	AATKSPQPT_PGR	VGSPQPSSSGGEK
G. gorilla	AATKSPQPT_PGR	AGSPQPSSSGGKK
P. troglodytes	AATKSPQPT_PGR	VGSPQPSSSGGEK
S. boliviensis	AATQSPQPT_PGR	AGSPQPSSSRGEK
S. scrofa	AATQTPHST_SRL	AGSPQPGNSQGKK
A. melanoleuca	AATHSPHPTVSGP	AGSPRPGNSQDKK
B. acutorostrata	AATQSPHPP_SRL	AGNSQPGTRQGKK
F. catus	AATHSRHPPLSGP	AGSPQPGDSQGKR
M. musculus	AATQNTQSM_PKQ	SQSPKS I NSKGRK
	SP/TP motif	SP motif

Supplementary Figure 1 Identification of phosphorylation sites in RECQL4 by mass spectrometry. **(a)** Coomassie Blue staining of 3XFLAG-tagged RECQL4 immunoprecipitated from HEK293T cells. 3XFLAG-tagged RECQL4 was expressed in HEK293T cells and purified from denatured cell lysate with M2 FLAG beads. Immunoprecipitated proteins were separated with SDS-PAGE and visualized with Coomassie Blue staining. **(b)** Annotation of MS/MS spectra for identification of phosphorylation sites Ser89 and Ser251. **(c)** Annotation of MS/MS spectra for identification of phosphorylation sites Ser27. **(d)** Sequence alignment of human RECQL4 peptides containing Ser89 and Ser251 with homologs in other mammals. The sequences were obtained from KEGG, and analyzed with Cluster W with default parameters. *H. sapiens*, human; *G. gorilla*, gorilla; *P. troglodytes*, chimpanzee; *S.*

boliviensis, black-capped squirrel monkey; *S. scrofa*, wild boar; *A. melanoleuca*, giant panda; *B. acutorostrata*, minke whale; *F. catus*, domestic cat; *M. musculus*, mouse.

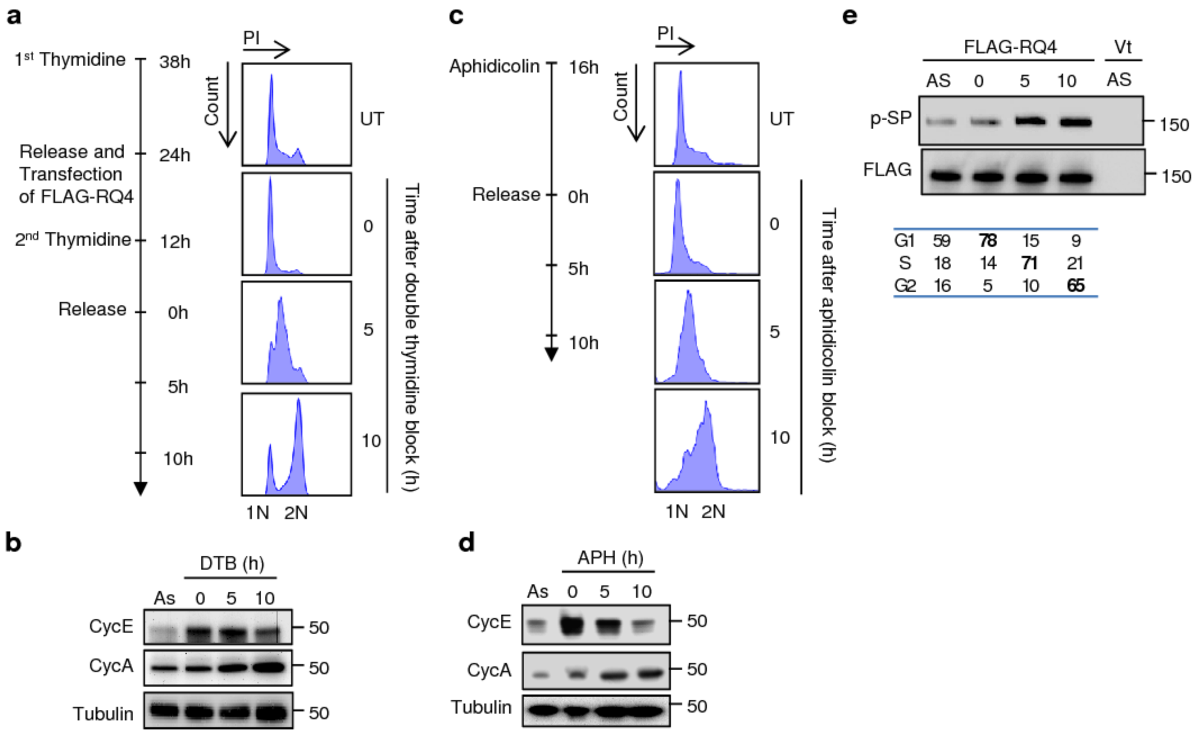

Supplementary Figure 2 CDK-mediated phosphorylation of RECQL4 takes places in the S and G2 phases of the cell cycle. **(a)** Flow cytometry analysis of cell cycle distribution of U2OS cells by propidium iodide staining. Schematic diagram of cell cycle synchronization by double thymidine block in U2OS cells. **(b)** Expression level of Cyclin A and Cyclin E in synchronized U2OS cells. **(c)** Schematic diagram of cell cycle synchronization by aphidicolin treatment and flow cytometry analysis of cell cycle distribution of HEK293T cells by propidium iodide staining. **(d)** Western blots showing protein levels of Cyclin A and Cyclin E in aphidicolin-treated HEK293T cells. **(e)** Cell cycle-dependent phosphorylation of RECQL4 on SP sites in HEK293T cells. HEK293T cells were transfected with the plasmid expressing 3XFLAG-RECQL4 or vector, and the cell cycle progression was synchronized with aphidicolin as shown in Panel c and d. The percentage of cells in the different phases of the cell cycle is listed below. 3XFLAG-RECQL4 was immunoprecipitated and analyzed by Western blotting with anti-p-SP antibody and anti-FLAG antibody.

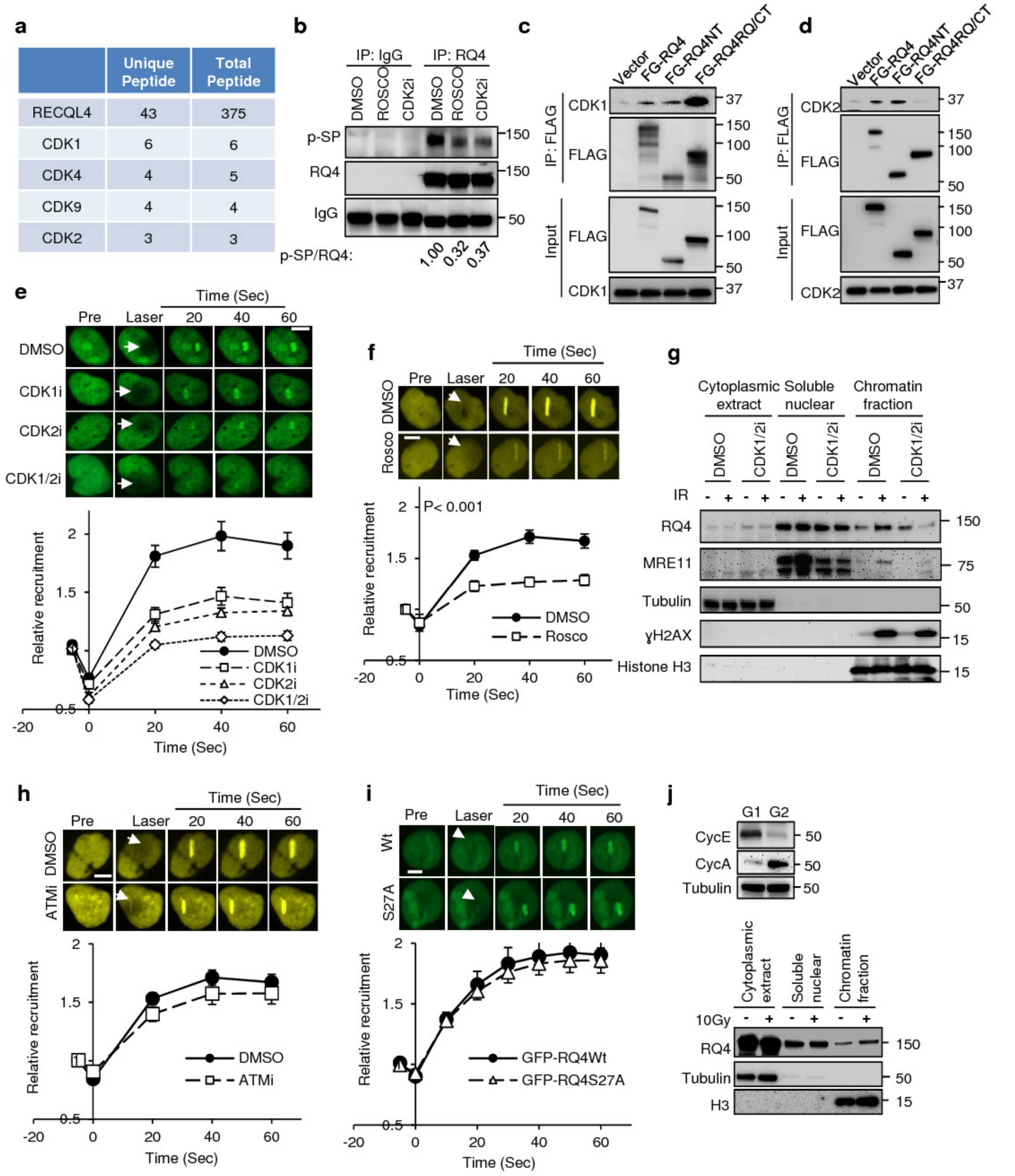

Supplementary Figure 3 CDK-mediated phosphorylation promotes RECQL4 recruitment to DSBs. **(a)** RECQL4-interacting CDKs were identified by mass spectrometry. GFP-tagged RECQL4 was immunoprecipitated from HEK293T cells with its interacting partners and subjected to mass spectrometry analysis. **(b)** Phosphorylation on SP motif of RECQL4 was repressed by CDK inhibitors. 20 μM roscovitine (ROSCO) or 20 μM CDK2i-II (CDK2i) were used for treated U2OS cells. **(c, d)** Mapping the interacting domains of RECQL4 with CDK1 **(c)** or CDK2 **(d)**. 3XFLAG-tagged RECQL4, RQ4NT (1-447AA) and RQ4RQ/CT (447-1208 AA) were purified from HEK293T cells with their interacting partners. CDK1 and CDK2 were detected with the related antibodies. **(e)**

Ser89/Ser251 phosphorylation promotes recruitment of RECQL4 to laser-induced DSBs. The relative intensities of the GFP signals are presented as mean ± s.e.m. The number of cells were quantified, for RQ4Wt, n=40; RQ4-2A, n=58; RQ4-2D, n=26. Scale bar, 5 µm. **(f)** Treatment with a pan-CDK inhibitor, Roscovitine, represses recruitment of YFP-RECQL4 to DSBs in HeLa cells. HeLa cells expressing YFP-RECQL4 were pretreated with DMSO or 20 µM Roscovitine for 4 hrs, and then submitted for micro-point laser irradiation. Relative intensities of YFP-RECQL4 are shown as mean ± s.e.m with p-values calculated by Student's t-test. Number of cells quantified: DMSO, n=17; Rosco, n=6. Scale bar, 5 µm. **(g)** CDK1 and CDK2 inhibition decreased accumulation of RECQL4 and MRE11 on chromatin in U2OS cells after IR stress. U2OS cells were pretreated with 10 µM RO3306 and 10 µM CDK2i-III for 4 hrs before10 Gy IR then subcellular fractions were prepared as described in Material and Methods. **(h)** Treatment of HeLa cells with the ATM selective inhibitor KU55933 does not significantly alter the recruitment of YFP-RECQL4 to DSBs. HeLa cells expressing YFP-RECQL4 were pretreated with DMSO or 20 µM ATMi (KU55933) for 4 hrs and then subjected to micro-point laser irradiation. The relative intensities of YFP-RECQL4 are shown as mean ± s.e.m. The number of cells analyzed were: DMSO, n=16; ATMi, n=8. **(i)** Substitution of Ser27 to alanine does not alter recruitment of RECQL4 to DSBs. GFP-tagged RECQL4 and RQ4S27A were expressed in U2OS cells, and real-time recruitment was observed and quantified. The relative intensities of GFP-RECQL4 and GFP-RQ4S27A are shown as mean ± s.e.m. The number of cells quantified: RQ4Wt, n=17; for RQ4S27A, n=24. Scale bar, 5 µm. **(j)** FLAG-RECQL4-2D is recruited to chromatin after IR stress in G1 U2OS cells. U2OS cells with endogenous RECQL4 depleted were transfected with 3XFLAG-RECQL4-2D, and synchronized with aphidicolin. Then subcellular fractions were prepared from the G1 cells with IR or without IR.

a

	Unique Peptide	Total Peptide
RECQL4	43	375
DDB1	37	49
CUL4A	3	3
DCAF7*	8	8
WDR77*	5	6
WDR61*	4	4
WDR82*	4	4
WDR5*	3	4
WDR26*	3	3
WDR92*	3	3
WDR75*	3	3
UBA1	3	3

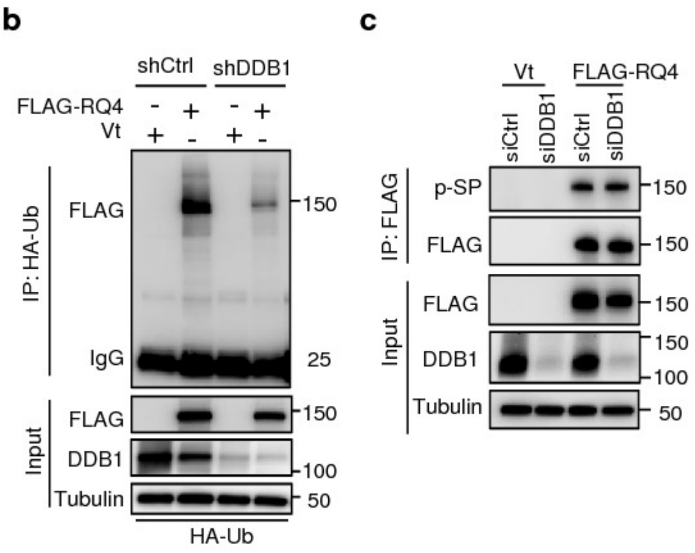

Supplementary Figure 4 The DDB1-CUL4A E3 ubiquitin ligase interacts with RECQL4 and ubiquitinates it in HEK293T cells. **(a)** Identification of DDB1, CUL4A and WD40-containing proteins (labeled with asterisks) in GFP-RECQL4 IP product from HEK293T cells by mass spectrometry. **(b)** Depletion of DDB1 reduces ubiquitination of RECQL4 in HEK293T cells. The plasmids expressing HA-tagged ubiquitin, 3XFLAG-tagged RECQL4 or vector were co-transfected into DDB-depleted or control cells, and ubiquitinated proteins were immunoprecipitated by HA-IP. Ubiquitinated 3XFLAG-RECQL4 was detected with anti-FLAG antibody by Western blotting. **(c)** Depletion of DDB1 does not alter phosphorylation of SP sites in RECQL4 in HEK293T cells. 3XFLAG-RECQL4 was isolated from DDB1-depleted and control cells and analyzed by Western blotting with anti-p-SP antibody.

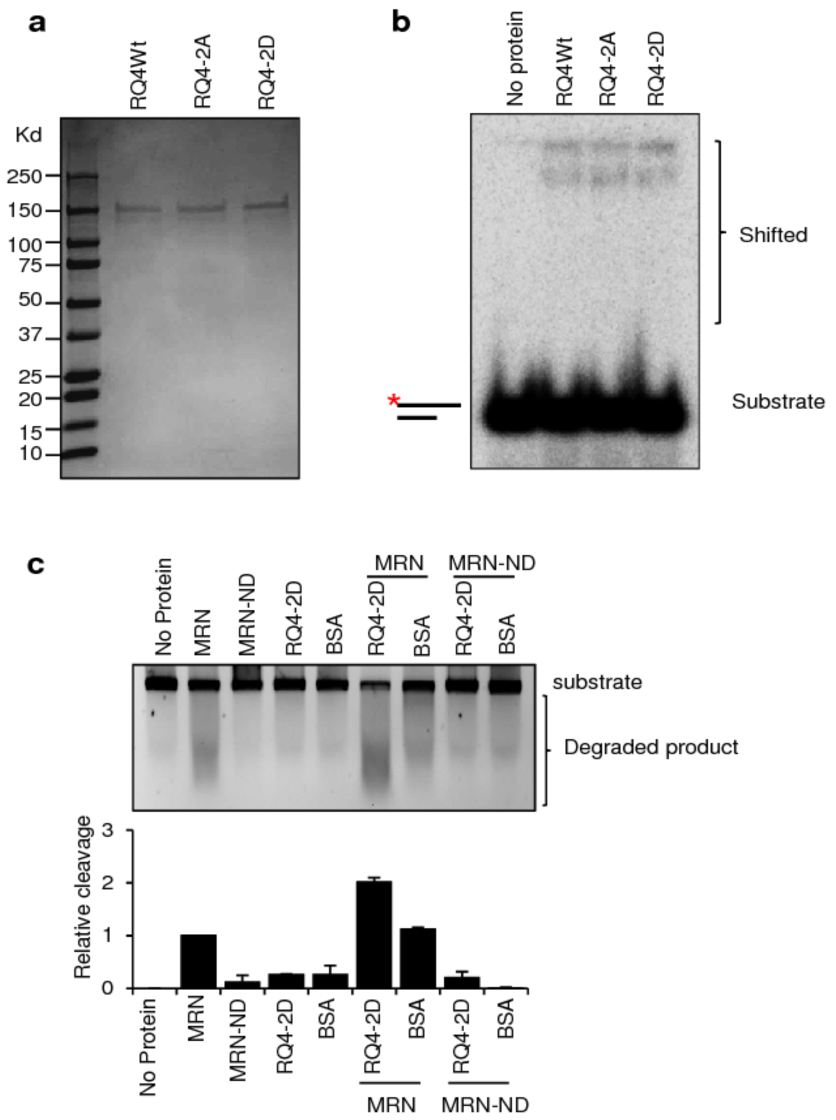

Supplementary Figure 5 Phosphorylation on Ser89/Ser251 does not affect stimulation of RECQL4 on nuclease activity of MRN. **(a)** Coomassie blue stained protein gel with purified 3XFLAG-tagged RQ4Wt, RQ4-2A and RQ4-2D. The plasmids expressing 3XFLAG-tagged RQ4Wt, RQ4-2A or RQ4-2D were transfected into HEK293T cells, and the proteins were purified 24 hrs later in the presence of phosphatase and protease inhibitor. For each sample, 1 µg of protein was loaded onto the SDS-PAGE gel and the purity of the purified proteins was visualized by Coomassie blue staining. **(b)** Analysis of DNA binding activity of RQ4Wt, RQ4-2A and RQ4-2D on the duplex DNA with 3' ssDNA overhang by gel shift mobility assay. 50 nM RECQL4 protein was applied. **(c)** Nuclease activity of MRN was stimulated by both RECQL4-2D in vitro. 20 nM MRN was mixed with 20 nM phosphor-mimetic mutant RQ4-2D in nuclease reaction buffer containing 100 ng closed circle phix174 ssDNA.

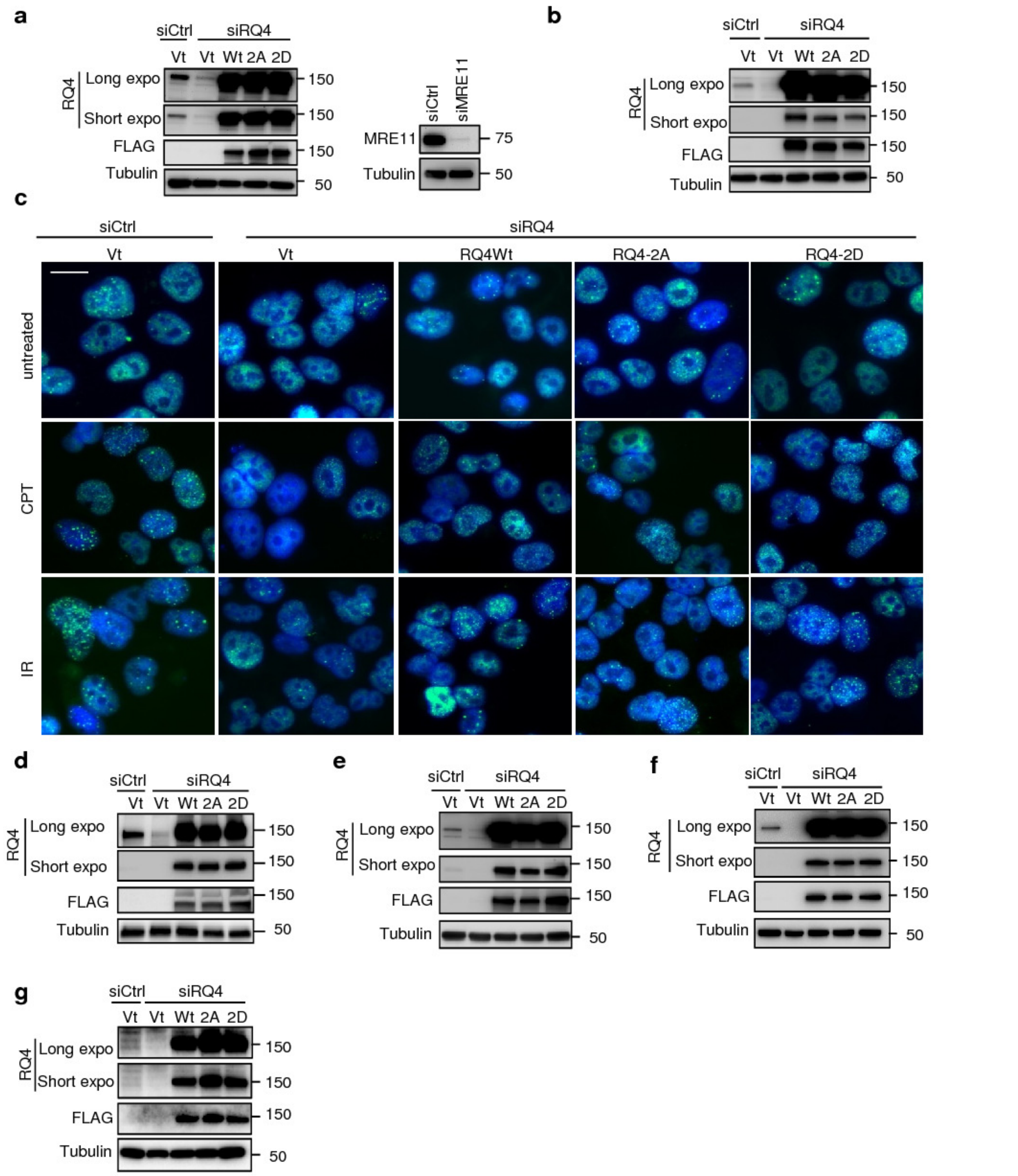

Supplementary Figure 6 Western blotting analysis of protein levels of endogenous RECQL4 and 3XFLAG-tagged RECQL4 WT or mutants: **(a)** in AID-DIvA U2OS cells used for DNA end resection assay and **(b)** U2OS cells used for RPA foci analysis. Representative images of RPA foci in U2OS cells after IR or CPT treatment are showed in **(c).** Scale bar, 20 µm. Western blotting analysis of protein levels in: **(d)** DR-GFP U2OS cells used for HR assay, **(e)** EJ5 U2OS used for NHEJ assay, **(f)** U2OS cells used for cell survival analysis, and **(g)** human fibroblasts used for SA-β-gal staining.

Supplementary Figure 7

Fig. 1a

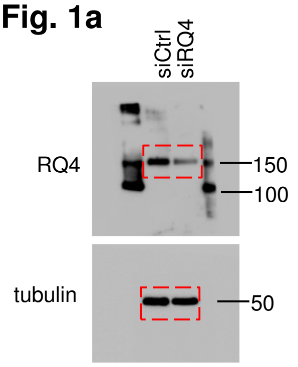

Fig. 1b

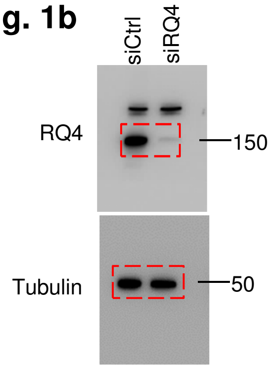

Fig. 1f

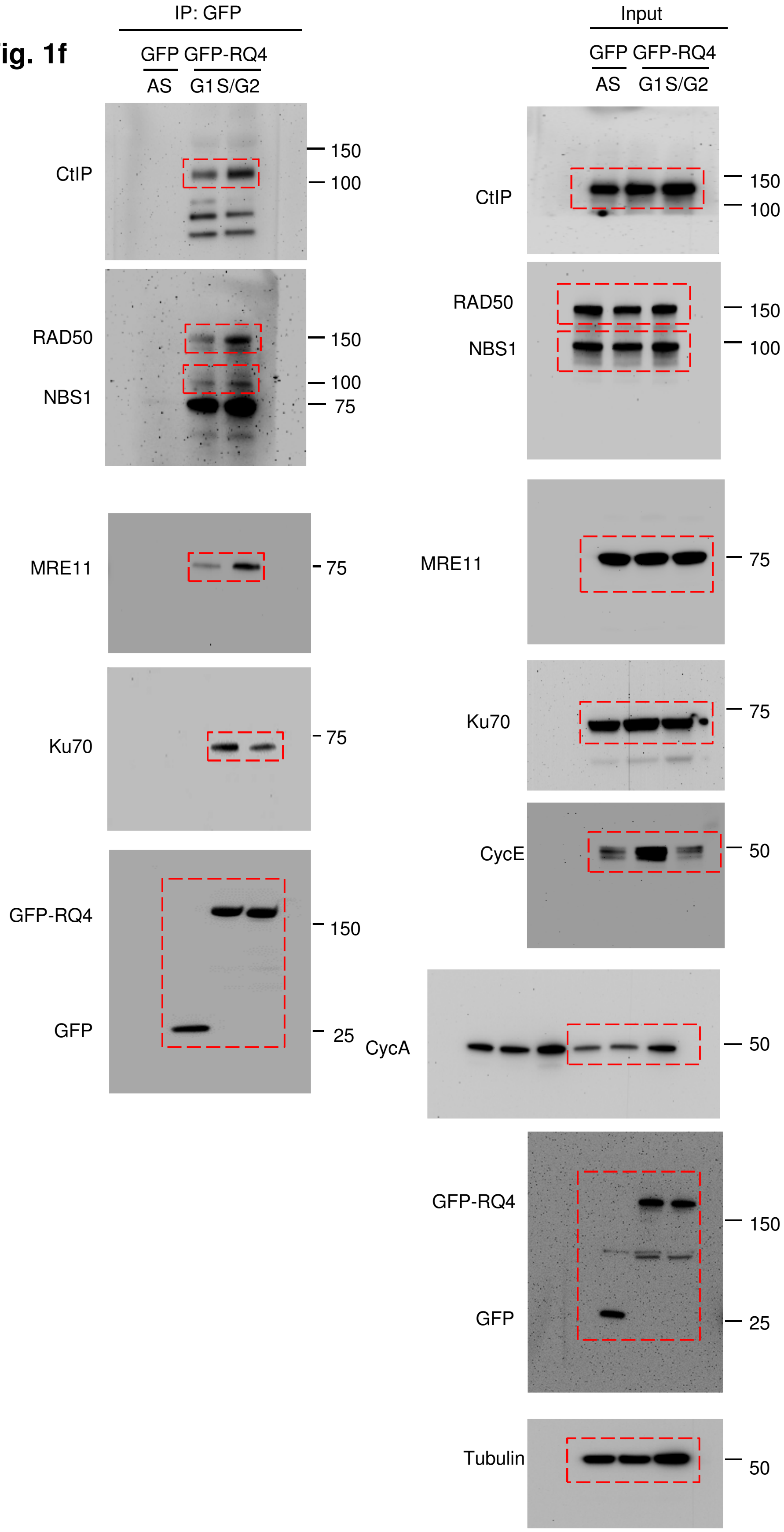

Supplementary Figure 7

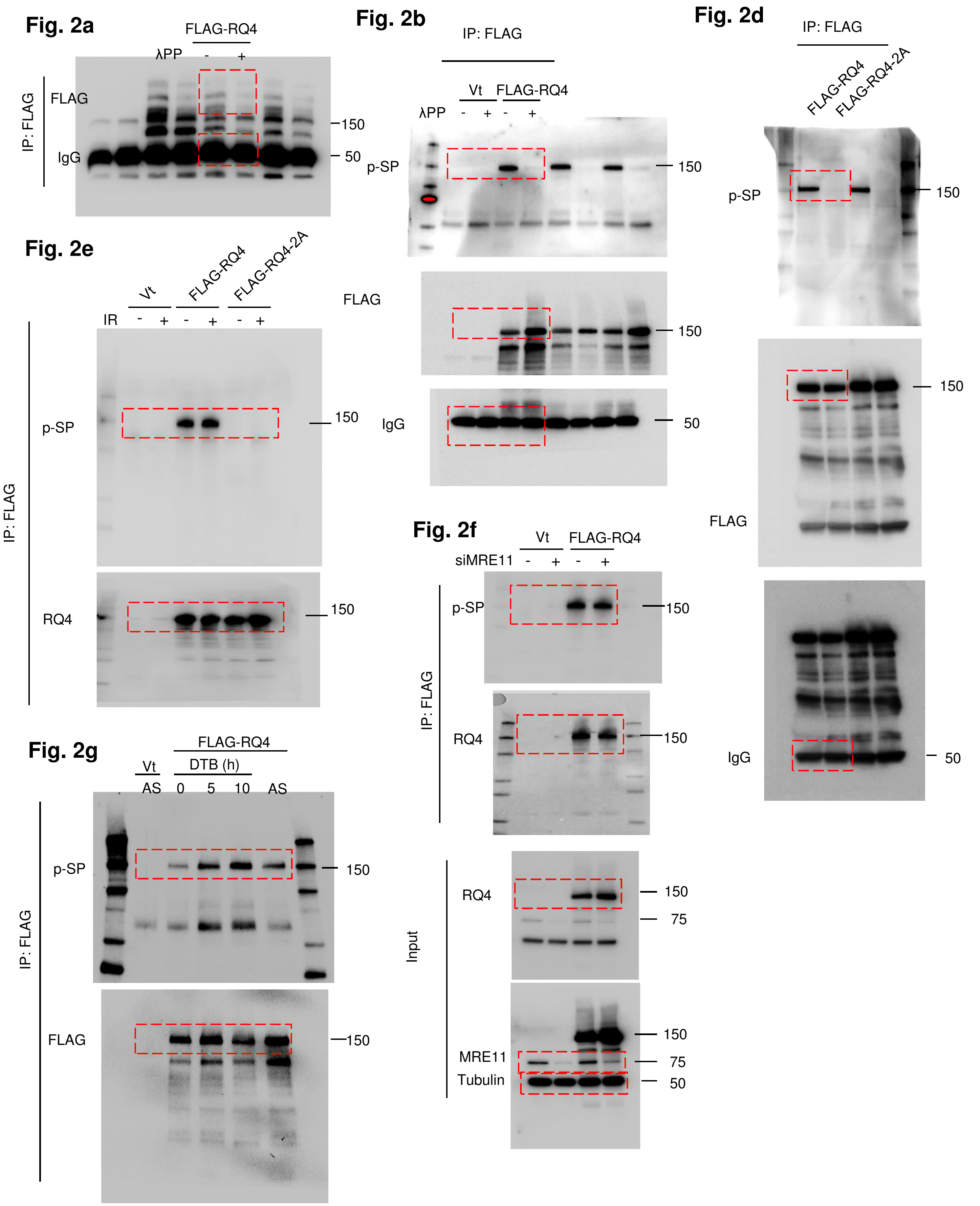

Supplementary Figure 7

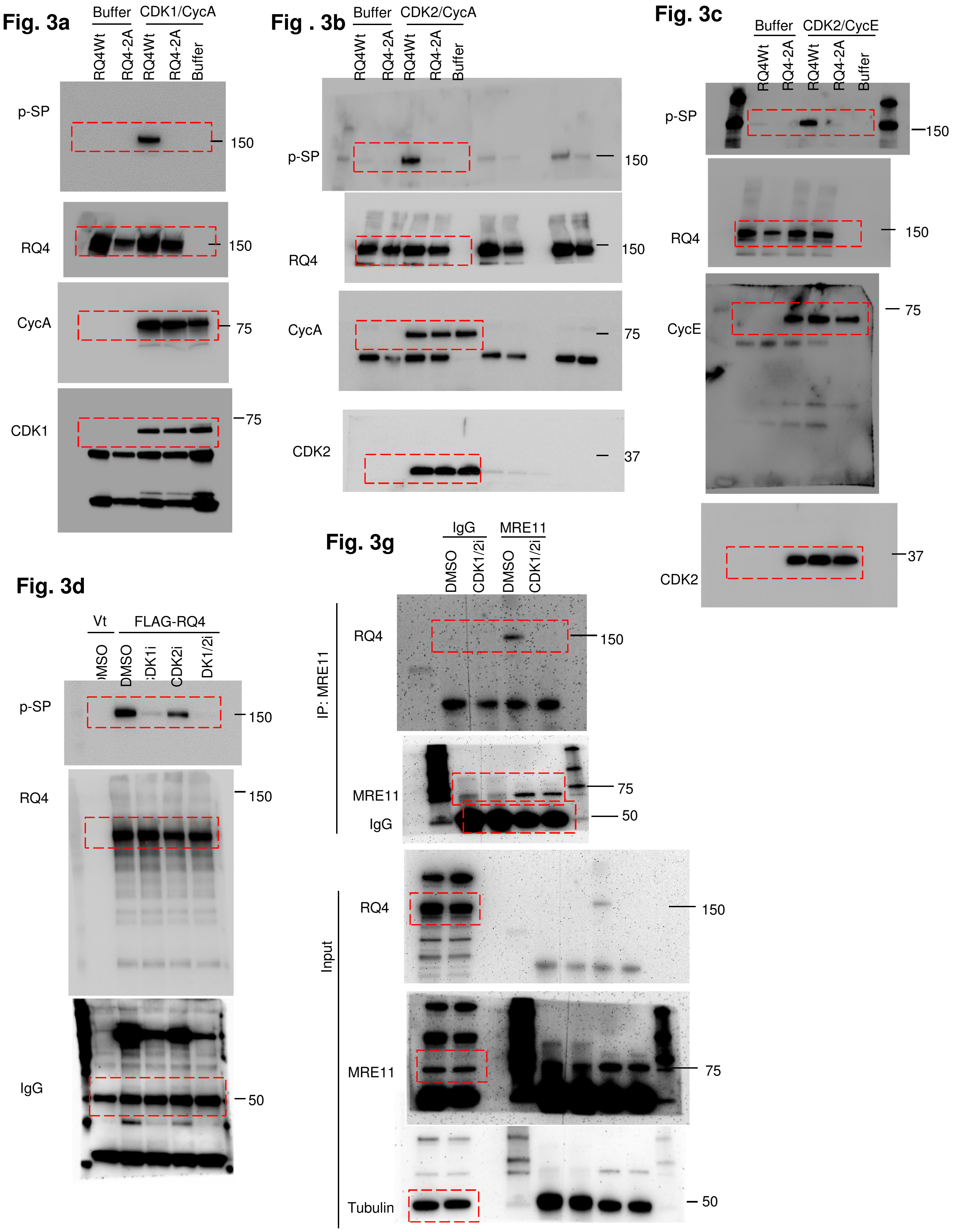

Supplementary Figure 7

Fig. 3f

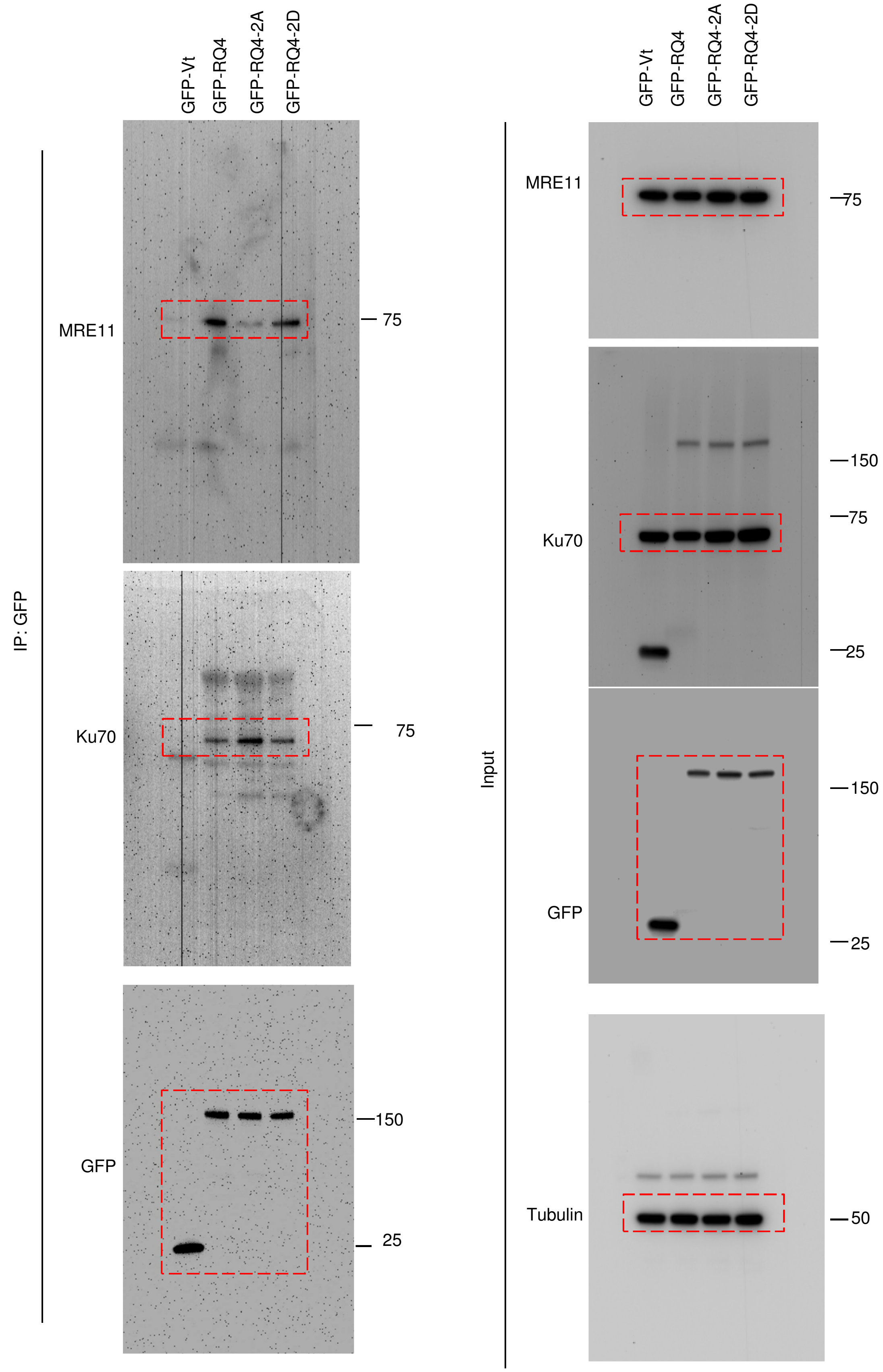

Supplementary Figure 7

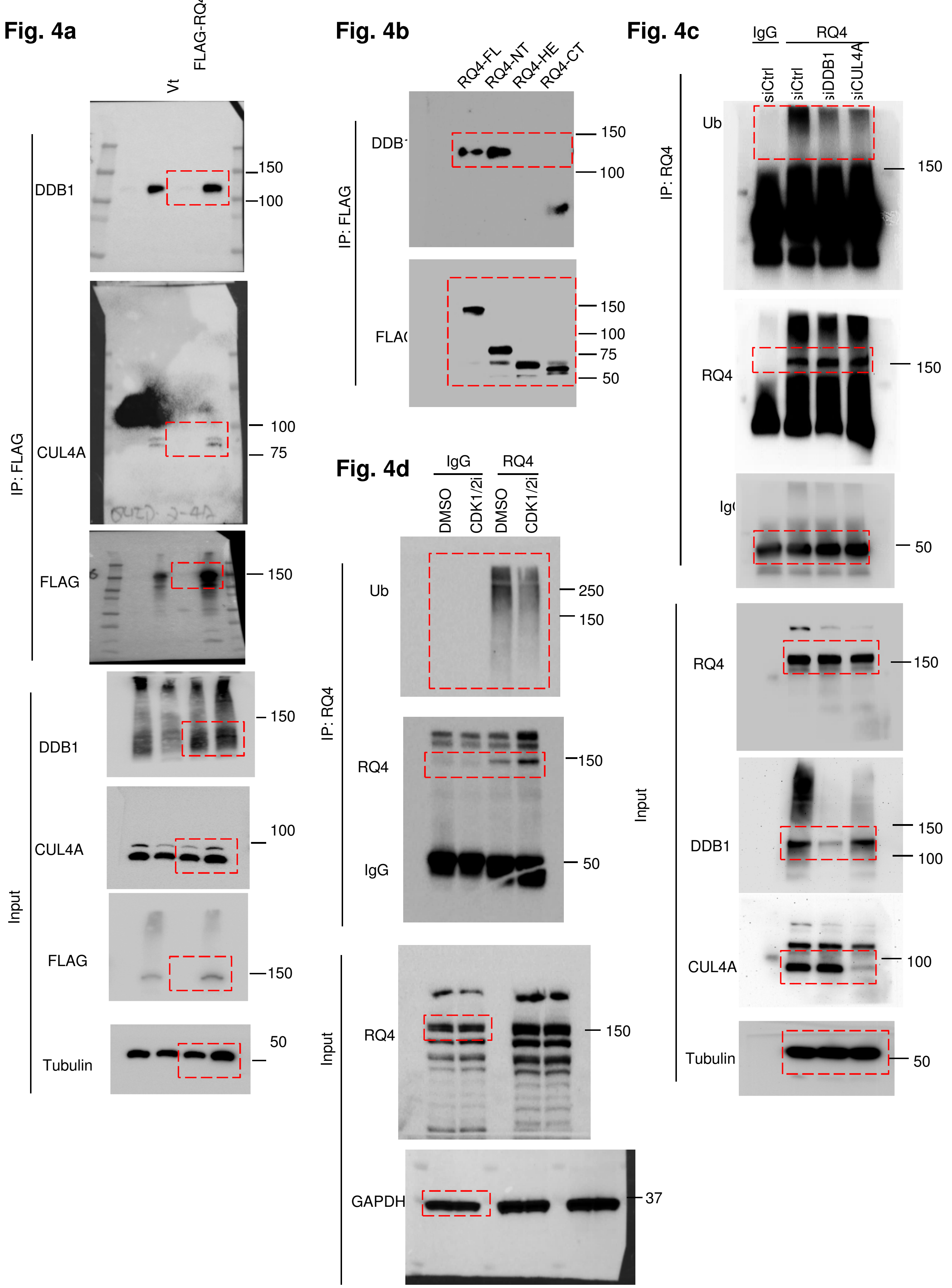

Supplementary Figure 7

Fig. 4e

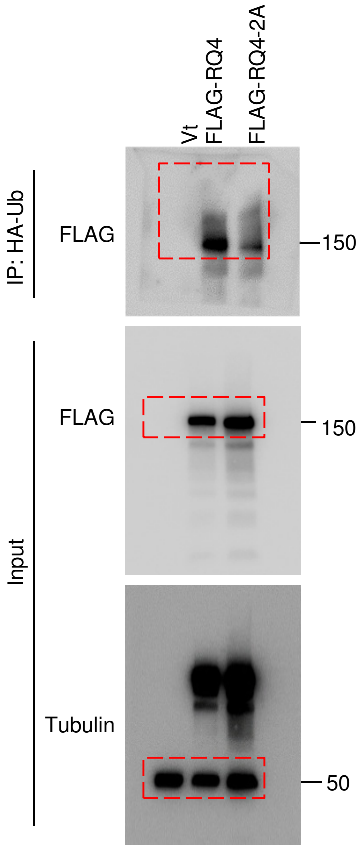

Fig. 4f

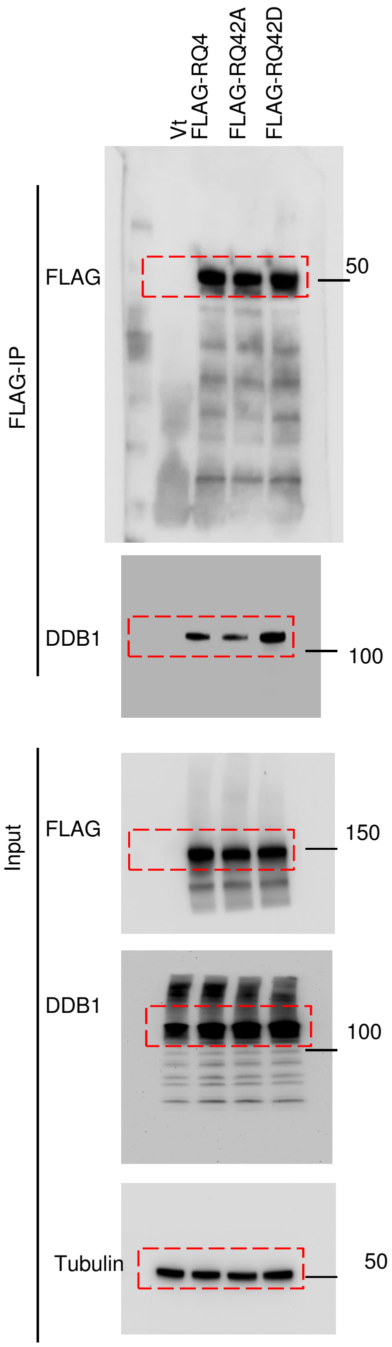

Fig. 4g

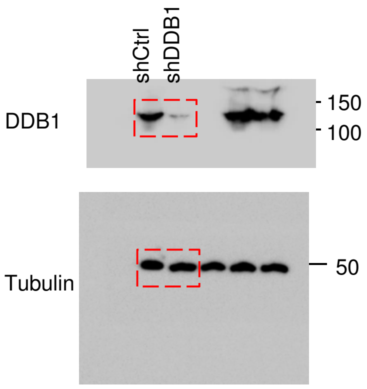

Fig. 4h

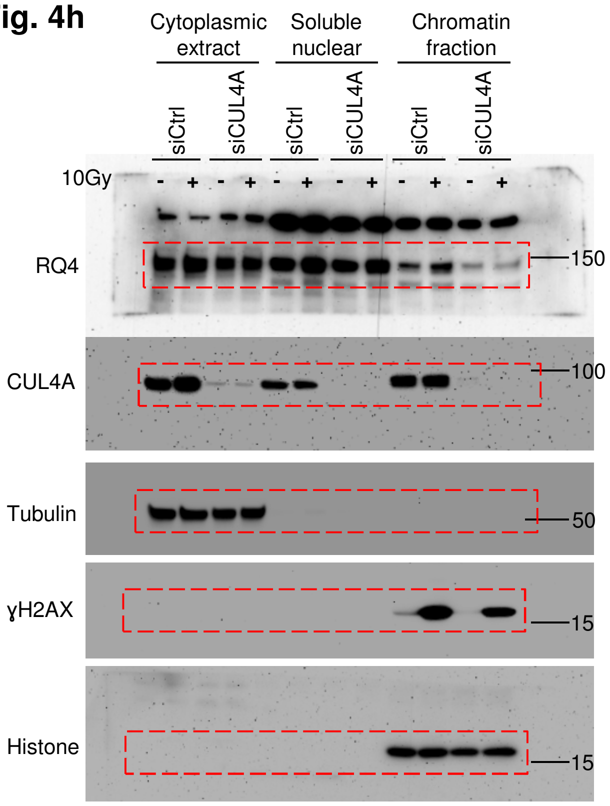

Supplementary Figure 7

Fig. S2b

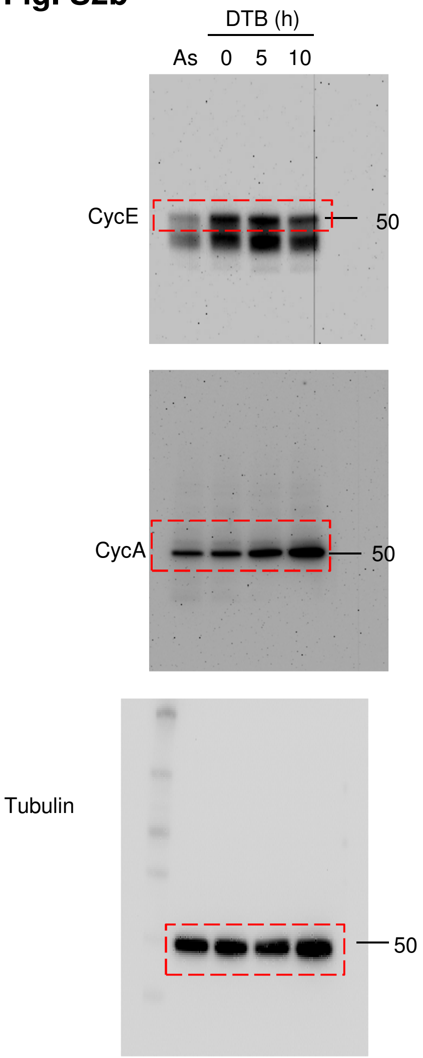

Fig. S2d

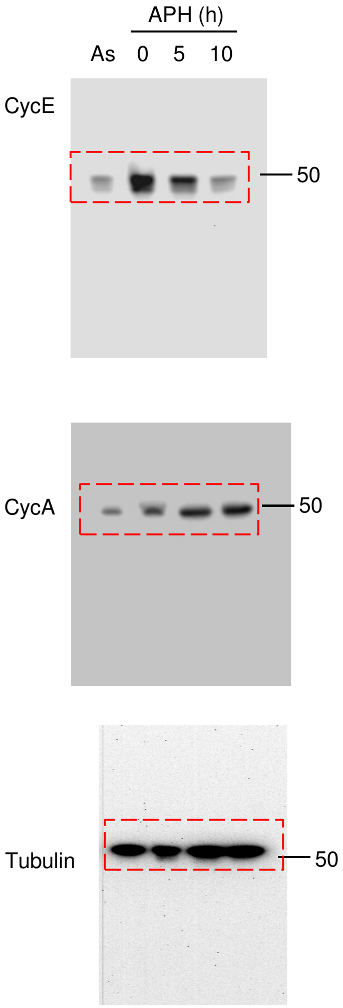

Fig. S2e

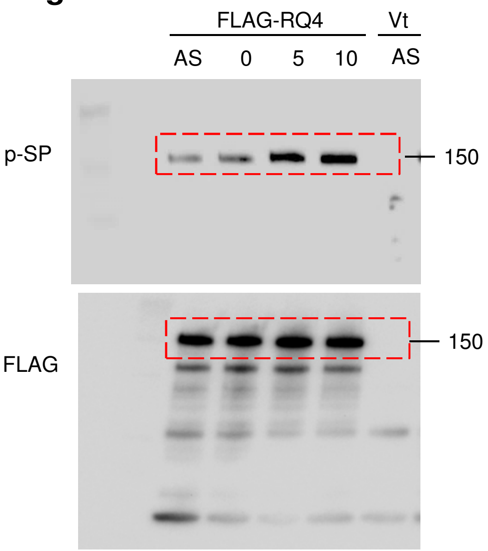

Supplementary Figure 7

Fig. S3b

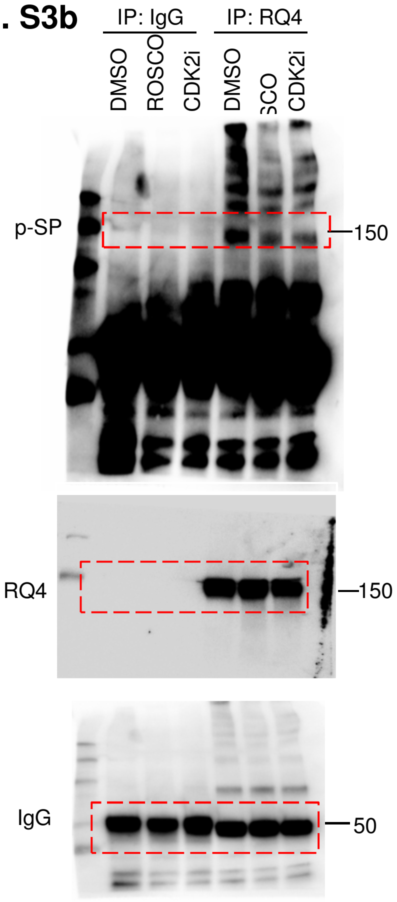

Fig. S3c

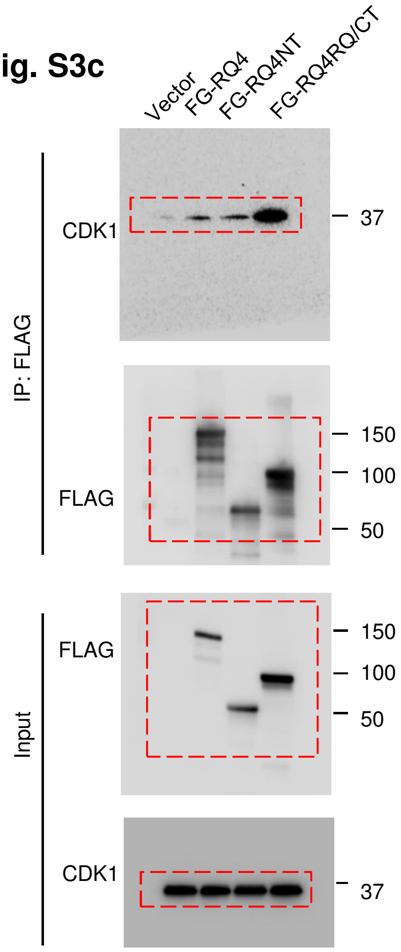

Fig. S3d

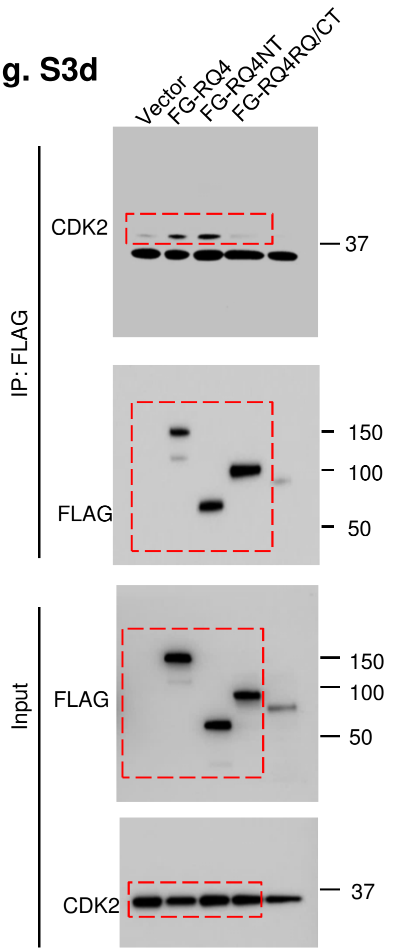

Fig. S3g

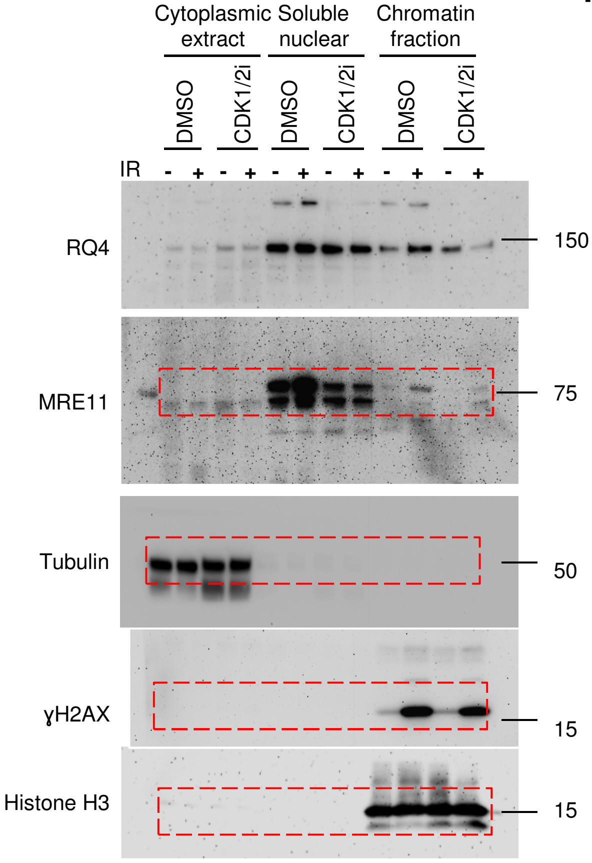

Fig. S3j

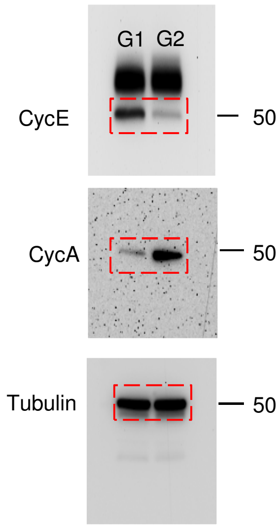

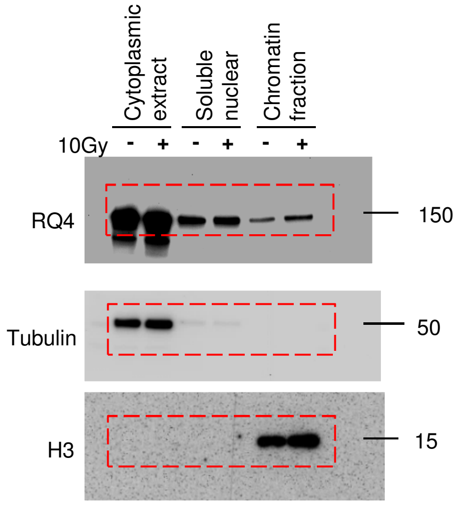

Supplementary Figure 7

Fig. S4b

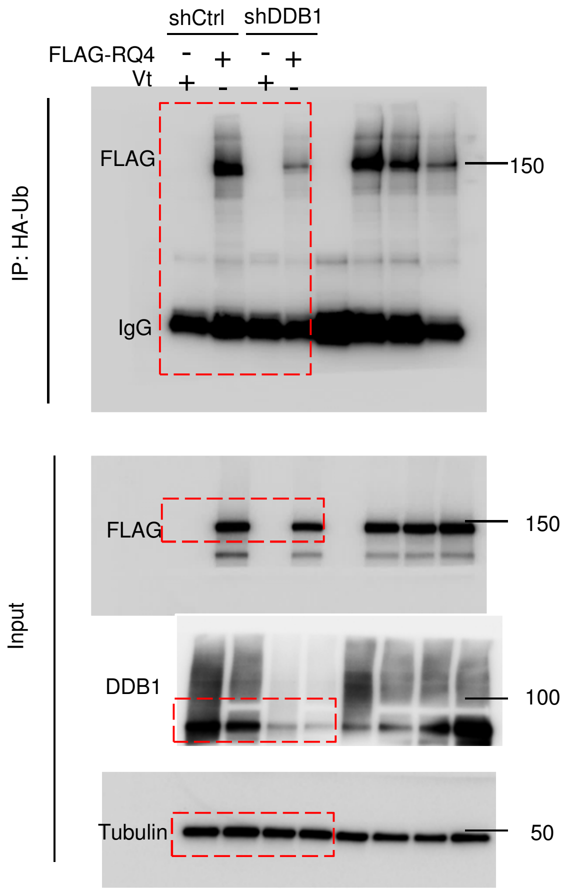

Fig. S4c

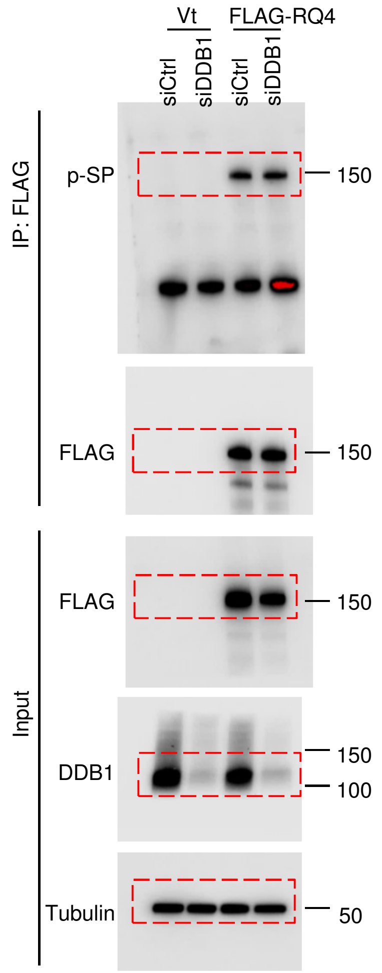

Supplementary Figure 7

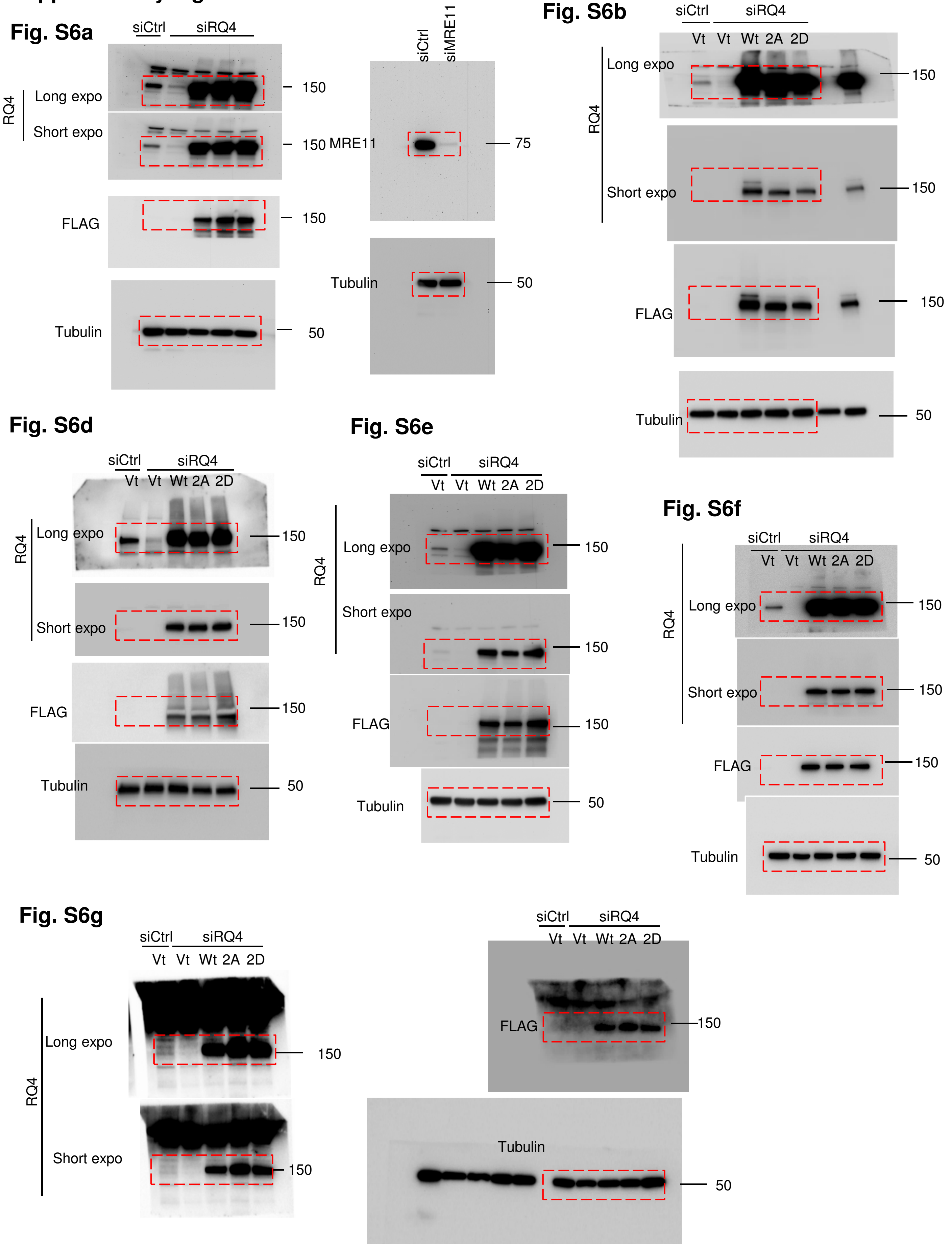

Supplement: Supplementary file 1 — Supplementary Information [file 41467_2017_2146_MOESM1_ESM.pdf]
