# Supplementary material for: Cell cycle-dependent phosphorylation regulates RECQL4 pathway choice and ubiquitination in DNA double-strand break repair
Source: Nat Commun. 2017 Dec 11;8:2039. doi: 10.1038/s41467-017-02146-3 (PMC5725494; doi:10.1038/s41467-017-02146-3)
Supplement: Supplementary file 3 — Description of Additional Supplementary Files [file 41467_2017_2146_MOESM3_ESM.docx]

**Description of Additional Supplementary Files**

File Name: Supplementary Data 1

Description: Identification of the proteins pulled down with RECQL4 by mass spectrometry
